# Responses to chemical cross-talk between the *Mycobacterium ulcerans* toxin, mycolactone, and *Staphylococcus aureus*

**DOI:** 10.1038/s41598-021-89177-5

**Published:** 2021-06-03

**Authors:** Laxmi Dhungel, Lindsey Burcham, Joo Youn Park, Harshini Devi Sampathkumar, Albert Cudjoe, Keun Seok Seo, Heather Jordan

**Affiliations:** 1 Department of Biological Sciences, Mississippi State University, P.O. Box GY, Starkville, MS 39762 USA; 2 Georgia State University, Atlanta, GA USA

**Keywords:** Microbiology, Pathogens

## Abstract

Buruli ulcer is a neglected tropical disease caused by the environmental pathogen, *Mycobacterium ulcerans* whose major virulence factor is mycolactone, a lipid cytotoxic molecule. Buruli ulcer has high morbidity, particularly in rural West Africa where the disease is endemic. Data have shown that infected lesions of Buruli ulcer patients can be colonized by quorum sensing bacteria such as *Staphylococcus aureus, S. epidermidis,* and *Pseudomonas aeruginosa*, but without typical pathology associated with those pathogens’ colonization. *M. ulcerans* pathogenesis may not only be an individual act but may also be dependent on synergistic or antagonistic mechanisms within a polymicrobial network. Furthermore, co-colonization by these pathogens may promote delayed wound healing, especially after the initiation of antibiotic therapy. Hence, it is important to understand the interaction of *M. ulcerans* with other bacteria encountered during skin infection. We added mycolactone to *S. aureus* and incubated for 3, 6 and 24 h. At each timepoint, *S. aureus* growth and hemolytic activity was measured, and RNA was isolated to measure virulence gene expression through qPCR and RNASeq analyses. Results showed that mycolactone reduced *S. aureus* hemolytic activity, suppressed *hla* promoter activity, and attenuated virulence genes, but did not affect *S. aureus* growth*.* RNASeq data showed mycolactone greatly impacted *S. aureus* metabolism. These data are relevant and significant as mycolactone and *S. aureus* sensing and response at the transcriptional, translational and regulation levels will provide insight into biological mechanisms of interspecific interactions that may play a role in regulation of responses such as effects between *M. ulcerans*, mycolactone, and *S. aureus* virulence that will be useful for treatment and prevention.

## Introduction

Buruli ulcer (BU) disease is a necrotizing skin disease whose etiological agent is *Mycobacterium ulcerans* (MU). The major virulence factor of MU is mycolactone, a cytotoxic macrocyclic lipid encoded by a giant plasmid pMUM001 that was acquired during emergence from an *M. marinum* progenitor^1^. Mycolactone leads to detachment and death of fibroblasts, epithelial cells, endothelial cells and karatinocytes, following exposure of 24–48 h, and leads to local and systemic immunomodulation^2^. Mycolactone’s mechanism on host cells has been shown to profoundly inhibit several host cytokines and chemokines that are dependent on Sec61 mediated translocation such as IL-17, TNFα, IL-6, chemokines IL-8 and MIP2, and adhesion molecules such as L-selectin. But other cytokines such as IL-1α, chemokines CCCL1, CXCL5 and adhesion molecules such as P-selectin, E-selectin, intracellular adhesion molecule (ICAM1) and lymphocyte function-associated antigen 1 are less affected^3,4^. Also, Interleukin-1β (IL-1β), a Sec-independent cytokine released through the caspase activated pathway has been shown to be less inhibited by mycolactone, suggesting selective inhibition of inflammatory cytokines in BU disease^3,5,6^. In fact, a recent study showed that mycolactone induced IL-1β production by murine and human macrophages, possibly through mycolactone-mediated membrane disturbance and the production of reactive oxygen species that trigger NLRP3/1 inflammasome activation, leading to the release of active IL-1β^7^. And, mycolactone does not appear to directly affect neutrophils in BU disease as its toxicity toward neutrophils is observed only in high doses, and absence of neutrophils in BU wounds is mainly attributed to poor neutrophil chemotaxis toward MU^3,8^.

*Staphylococcus aureus, S. epidermidis and Pseudomonas aeruginosa* have been isolated from the infected lesions of BU patients^9–12^. However, disease pathology normally associated with these pathogens, such as pain, pus, or redness is strikingly absent in these ulcers, especially prior to MU antibiotic treatment^9,10,13^. Despite the lack of pathology, co-colonization by these pathogens may promote delayed wound healing, especially after the initiation of MU antibiotic therapy. *Staphylococcus aureus* is a primary cause of skin and soft tissue infections, prosthetic-joint infections and infective endocarditis^14–17^, but may also be human normal flora, particularly residing within anterior nares^18^. *S. aureus* expresses several factors required for colonization (adhesins) and invasion (coagulase, staphylokinase)^19^, lysis (hemolysins), immune evasion (leukotoxins), and increased pathogenicity (enterotoxins and TSST1), which are regulated by the SaeRS (*S. aureus* exoprotein expression) two component system (among others)^20–22^; but also by the accessory gene regulator system (Agr), that regulates virulence through quorum sensing mechanisms^23,24^.

What is less clear, then, is whether the absence of pus and other pathology in a co-infected wound from a BU patient is due to decreased virulence of *S. aureus* or immune suppression caused by mycolactone. *S. aureus* skin infection is typically characterized by abscess formation mediated by neutrophils, where the absence of pro-inflammatory cytokine IL-1β has been associated with increased lesion size in murine skin infections^4,25^. Also, *S. aureus* colonizing BU wounds contains virulence genes such as *hla* (hemolysin)*, sak* (staphylokinase), and *luk* (several leukocidins including *lukD*, *lukE*, *lukF*, *lukS*, and PVL) with a potential to cause serious and invasive infections. However, absence of any pus or invasive infection in BU patients colonized with *S. aureus* suggests attenuation of *S. aureus* virulence in BU disease and a potential role of mycolactone in driving this attenuation^9,26^.

The macrocyclic structure of mycolactone is similar to quorum sensing (QS) compounds in other bacteria. Studies have shown that some QS molecules such as 3-oxo-C_12_-Homoserine lactone produced by *P. aeruginosa* can inhibit QS systems of other bacteria, such as inhibition of *S. aureus sarA* and *agr* expression^27^*.* Auto-inducing peptides produced by *S. epidermidis* and *S. schleiferi* can also inhibit expression of Agr regulated virulence genes in *S. aureus*^20,28^. The inability of isolated pathogens such as *S. aureus* to cause disease in BU wounds is intriguing and suggest mechanisms of MU to attenuate the virulence of those pathogens during infection, contributing to niche competition, and predominance of MU in the wound. On the other hand, co-colonization in a MU wound could allow *S. aureu*s nutrient acquisition and a means of dispersal at a lowered energy expense. Therefore, the purpose of this study was to understand the effect of mycolactone on *S. aureus* growth, expression of virulence factors and hemolysis.

## Results

### S. aureus growth is not inhibited by mycolactone

**Figure 1:**
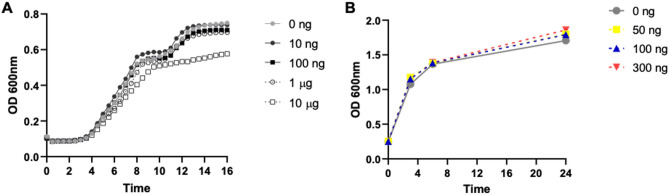
(**A**) Dose effect of mycolactone on *S. aureus* growth measured by OD600 for up to 16 h. (**B**) Comparison of OD600 of *S. aureus* incubated alone, or with mycolactone or EtOH at 3H, 6H, and 24H.

*Staphylococcus aureus* was incubated with mycolactone (concentration 0 ng–10 µg) over time to determine whether there was any effect by mycolactone on *S. aureus* growth. Based on optical density (OD600nm), low to moderate mycolactone concentrations (1 ng–1 µg) had no effect on *S. aureus* growth for up to 16H, though 10 µg mycolactone had some effect on S*. aureus* growth after 7H (Fig. 1A). We also measured corresponding *S. aureus* growth in association with our gene expression experiments across timepoints. There was no statistical difference in growth between *S. aureus* containing 0, 50, 100 and 300 ng/mL mycolactone and control at 3H, 6H and 24H timepoints (Fig. 1B).

### Mycolactone reduces *S. aureus* hemolytic activity

**Figure 2:**
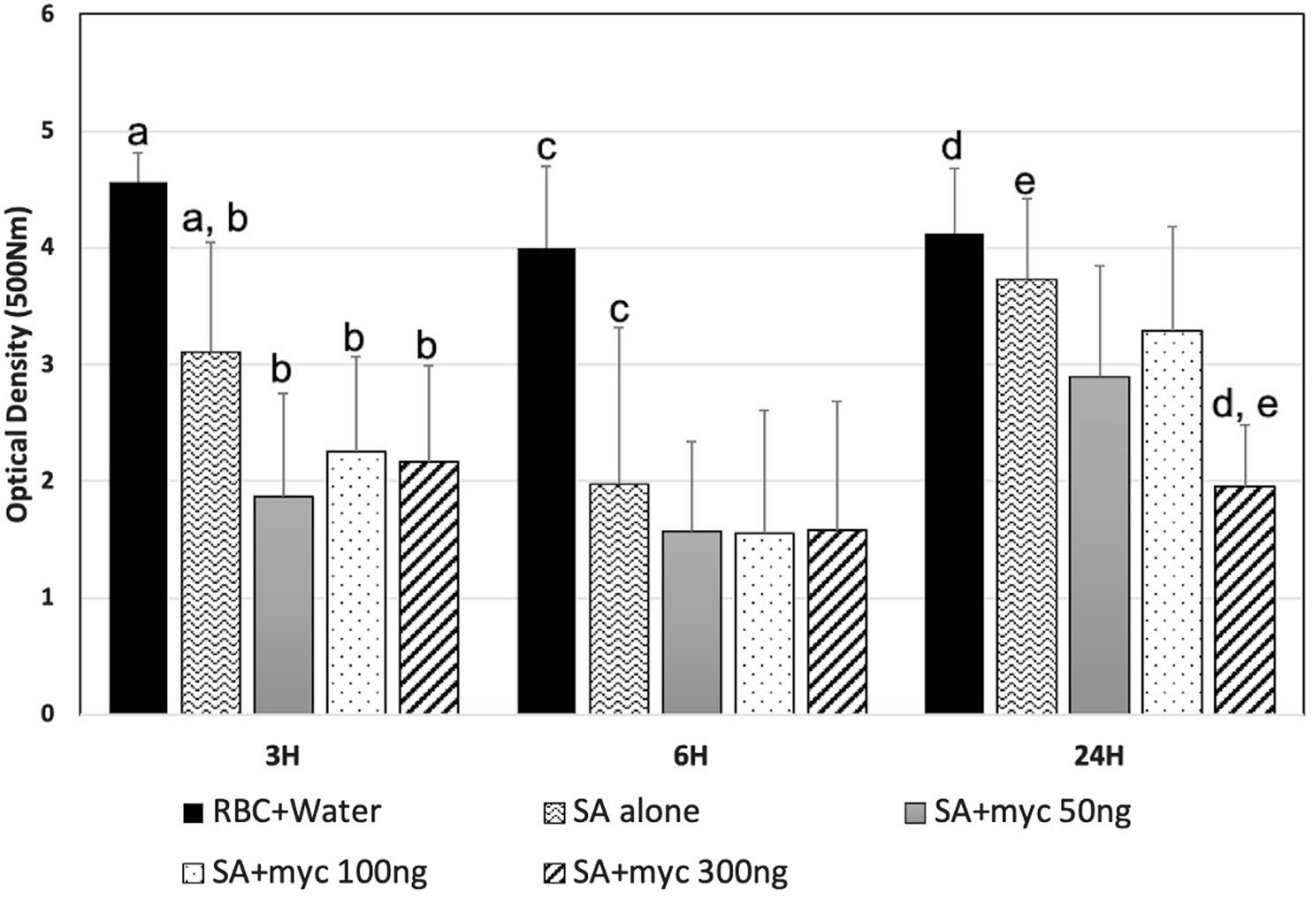
Mycolactone co-Incubation reduced *S. aureus* hemolysis over time. Graph showing OD500 of red blood cells incubated with water (positive control), *S. aureus* alone, *S. aureus* plus 50 ng mycolactone, *S. aureus* plus 100 ng mycolactone, or *S. aureus* plus 300 ng mycolactone. Significance was determined as < 0.05. (a–c), and (d,e) denotes significantly different treatments at 3H, 6H, or 24H, respectively.

*Staphylococcus aureus* hemolytic activity was significantly decreased for *S. aureus* incubated with mycolactone (concentration 50, 100 and 300 ng) compared to *S. aureus* incubated with the mycolactone vehicle control (EtOH) at 3H. While hemolysis was decreased for *S. aureus* incubated with all concentrations at 6H compared to controls, this decrease was not statistically significant. At 24H, hemolysis was only significantly reduced for *S. aureus* incubated with 300 ng mycolactone (Fig. 2).

### Mycolactone leads to modulation of *S. aureus* global regulator gene expression

**Figure 3:**
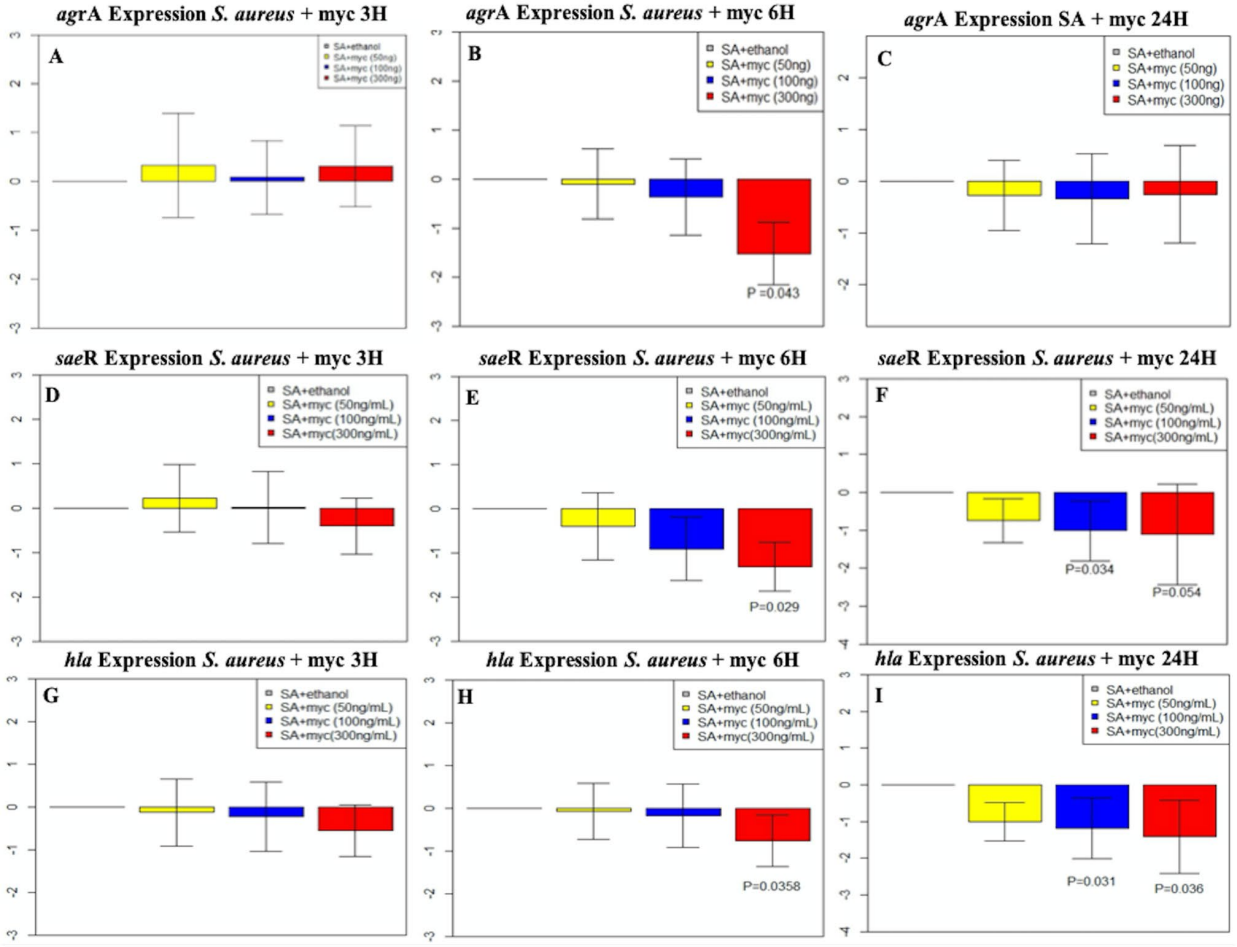
Gene expression of S*. aureus agr*A, *saeR*, and *hla* when exposed to 50, 100, or 300 ng mycolactone concentrations over time. (**A**–**C**) *agr*A regulation at 3H, 6H, and 24H, respectively. (**D**–**F**) *sae*R regulation at 3H, 6H, and 24H, respectively. (**G**–**I**) *hla* expression at 3H, 6H, and 24H respectively. Regulation was measured for all genes against the *aroE* housekeeping gene.

The effect of mycolactone on *S. aureus agrA*, *saeR* and *hla* virulence genes was determined by RT-qPCR. The results showed that *agrA* was not significantly modulated at 3H (Fig. 3A) but was significantly downregulated for *S. aureus* incubated with mycolactone (300 ng at 6H (p = 0.043, Fig. 3B). However, *agrA* returned to control levels at 24H (Fig. 3C). RT-qPCR of *sae*R gene activity showed no statistical difference from control at 3H (Fig. 3D), but, *sae*R was significantly downregulated at 6H for the 300 ng mycolactone treatment (p = 0.029, Fig. 3E) and at 24H (p = 0.04 for 100 ng and p = 0.006 for 300 ng, Fig. 3F). Similarly*,* the *hla* gene was not significantly different from control at 3H (Fig. 3G), but was significantly downregulated for *S. aureus* incubated with mycolactone (300 ng) at 6 h (p = 0.04, Figure H) and at 24 h (100 ng, p = 0.03 and 300 ng, p = 0.05, Fig. 3I) compared to control.

### Mycolactone suppresses hla promoter activity

**Figure 4:**
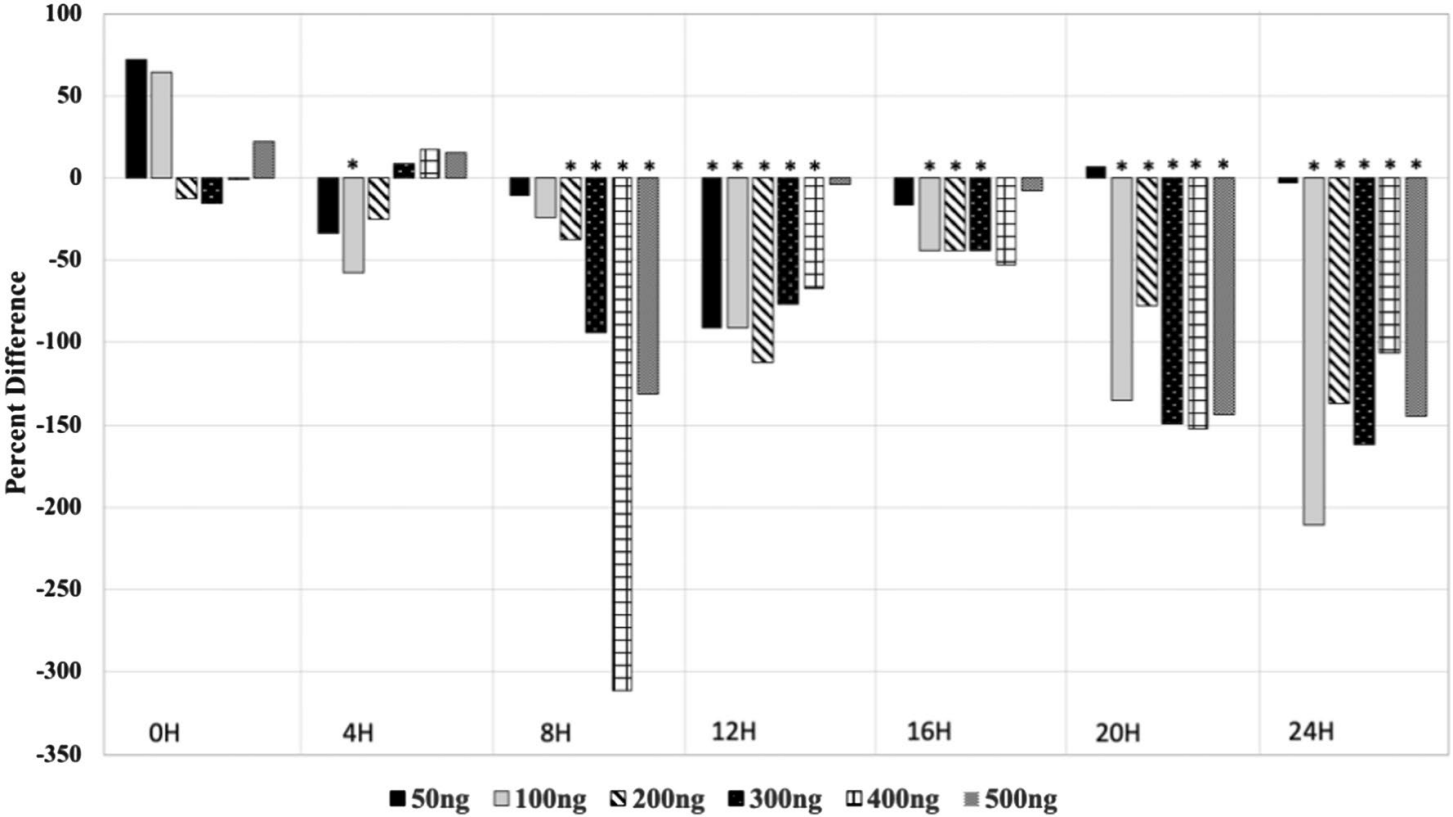
Percent difference in *hla* promoter activity (Luminescence) of mycolactone treatments from vehicle control.

We tested a range of mycolactone (50 to 500 ng) supplementation with *S. aureus* on promoter activity of the *hla* gene. Mycolactone at 100 ng was the minimal concentration to suppress *hla* gene expression across our tested timepoints. Significant suppression of *hla* promoter activity occurred between 3.5–6.5H, 9–12.5H, and 14–24H for the *S. aureus* + 100 ng mycolactone treatment (Supplemental Fig. S1). At 4 h, the mean percent difference in luminescence between the vehicle control and mycolactone treatment was − 57% (p = 0.004, Fig. 4). Luminescence in the mycolactone treatments continued to be lower than vehicle control at 8H (− 24%, p = 0.24), 12H (− 91%, p = 0.01), 16H (− 44%, p = 0.007), 20H (− 135%, p = 0.0008), and 24H (− 211%, p = 0.0002). *S. aureus* + 200 ng mycolactone significantly suppressed *hla* promoter activity between 2 and 13.5H, then between 15.5 and 24H (Supplemental Fig. S1). At 4H, the mean percent difference in luminescence between the mycolactone treatment and vehicle control was − 25% (p = 0.370). Luminescence in the mycolactone treatments remained less than that from the vehicle control for 8H (− 37%, p = 0.05), 12H (− 112%, p = 0.008), 16H (− 44%, p = 0.024), 20H (− 78%, p = 0.014), and 24H (− 137%, p = 0.001, Fig. 4). Mycolactone with 300 ng concentration suppressed *hla* promoter activity at 1.5H, between 6.5 and 12.5H, at 15H, and between 16 and 24H (Supplemental Fig. S1). The mean percent difference in luminescence between mycolactone treatment and vehicle control was 9% (p = 0.720) at 4H, − 94% (p = 0.03) at 8H, − 77% (p = 0.0008) at 12H, − 44% (p = 0.041) at 16H, − 149% (p = 0.004) at 20H, and − 162% (p = 0.001) at 24H. Mycolactone at 400 ng significantly suppressed *hla* promoter activity at 2H, between 7.5–8H, 9.5–12H, and 16.5–24H (Supplemental Fig. S1). Mean percent difference in luminescence of mycolactone treatments compared to control was 17% (p = 0.37) at 4H, − 311% (p = 0.02) at 8H, − 67% (p = 0.036) at 12H, − 53% (p = 0.101) at 16H, 152% (p = 0.007) at 20H, and − 106% (p = 0.008) at 24H. Finally, 500 ng mycolactone significantly suppressed *hla* promoter activity between 7.5 and 10.5H then 17 and 24H (Supplemental Fig. S1). Luminescence was 15% greater in mycolactone treatments than controls at 4H (0.54). But luminescence decreased in mycolactone treatments compared to controls at 8H (− 131%, p < 0.001), 12H (− 4%, p = 0.46), 16H (− 8%, p = 0.42), 20H (− 144%, p = 0.01), and 24H (− 145%, p = 0.02). Significance percent differences at specific timepoints is indicated in Fig. 4 with a star.

### Mycolactone modulates *S. aureus* global gene expression

To measure the effects of mycolactone on *S. aureus* global gene expression, we conducted a small-scale transcriptome study using RNASeq analysis. Sequencing and trimming yielded an average fragment length of 143, and between 53 and 101 million reads per sample. Altogether, 150 genes were significantly differentially regulated in the *S. aureus*-mycolactone treatments in comparison to the *S. aureus*-EtOH controls (Supplemental Fig. S2). Complete gene lists of significantly up-or down-regulated genes are shown in Supplemental Table S1. Ninety-two genes were significantly up-regulated and 58 genes were significantly downregulated in response to mycolactone in comparison to control.

#### Three hour timepoint

**Figure 5:**
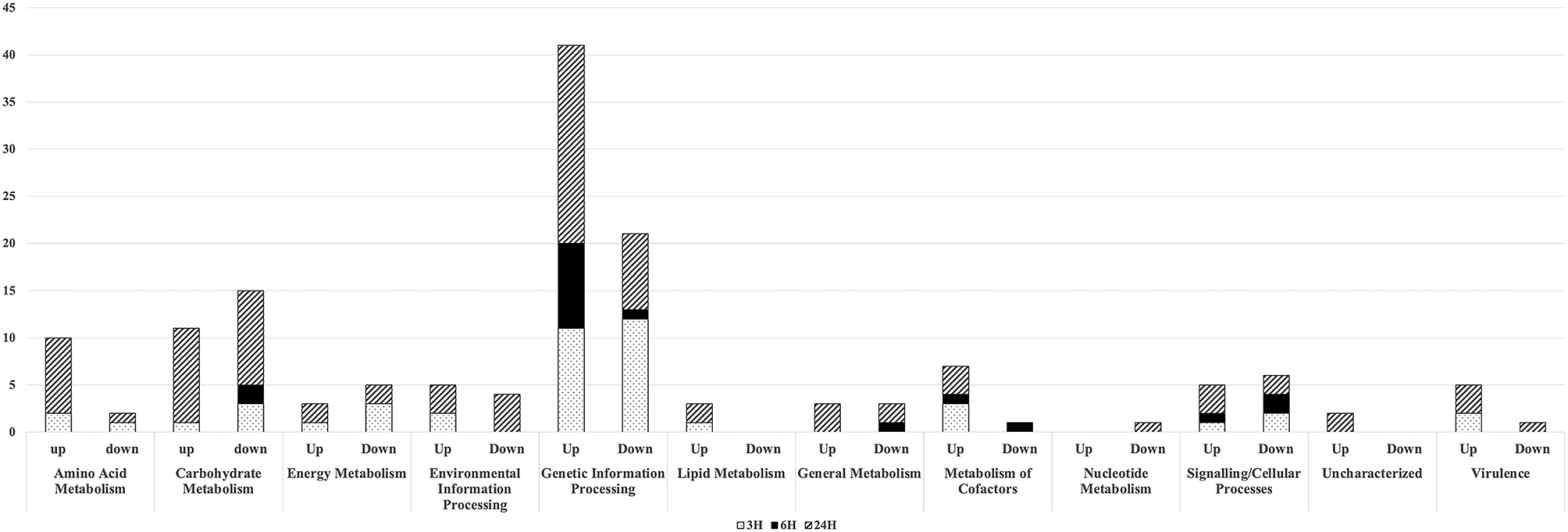
Number of genes significantly modulated in mycolactone treatments compared to controls according to functional category and timepoint.

Forty-four differentially regulated genes were identified at the 3H timepoint. These included 21 downregulated and 23 upregulated genes in the *S. aureus*-mycolactone treatment compared to control. Fourteen genes associated with metabolism were differentially regulated (Fig. 5). For instance, a thioredoxin reductase (*trxB*), involved in amino acid metabolism was downregulated, while two genes for arginine biosynthesis (*argG* and *argH*) were upregulated. One gene *fakb2*, was upregulated, and involved in lipid metabolism.

Three genes involved in metabolism of cofactors/vitamins were upregulated including genes involved in folate (*dfrA*), thiamine (*thiD*), and vitamin B6 (*pdxS*) metabolism. Carbohydrate metabolism was modulated where two genes, *adhE* and *adhP*, encoding for alcohol dehydrogenase were downregulated as was one gene for l-lactate dehydrogenase (LDH). The *ulaB* gene, encoding the PTS lactose transporter subunit IIB, was upregulated. Three genes for energy metabolism were downregulated including *qoxA*, *qoxB*, and *qoxC,* genes for probable quinol oxidase subunits. However, nitrate reductase beta chain *narH*, involved in nitrogen metabolism, was upregulated (Fig. 5).

Twelve genes involved in genetic information processing were downregulated at the 3H timepoint (Fig. 5). These included 7 genes involved in mechanistic components of translation, one for transcription, and three transcriptional regulators including the transcriptional activator *sarR*, a negative regulator of *sarA* transcription and positive regulator of expression of primary transcripts RNAII and RNAIII generated by the Agr locus. Genes encoding a universal stress protein (*uspA*) and a cold shock protein (*cspC*), along with the preprotein translocase subunit *secY* were also downregulated. Among those upregulated within the genetic information processing category included the transcriptional activator, *rinB*, *gtfB,* encoding a stabilizing protein that is part of the accessory SecA2/SecY2 system, the repair gene *recR*, and 8 genes involved in mechanistic components of translation.

Genes encoding a probable potassium-transporting ATPase B chain gene (*kdpB*) and a glutamine ABC transporter ATP-binding protein (*glnQ*), both involved in environmental information processing were upregulated. Also, the signaling and cellular processing genes encoding l-lactate permease (*lldP*) and xanthine permease (*pbuX*) were downregulated while *sspA*, encoding a V8-like Glu-specific serine protease was upregulated. Finally, surface protein A^29^ and clumping factor A (*clfA*), both involved in virulence, were upregulated at the 3 h timepoint.

#### Six hour timepoint

Eighteen genes were significantly modulated at the 6H timepoint, including six downregulated and twelve upregulated. These included downregulation of *adhE*, and also of *wecB*, a UDP-N-acetylglucosamine 2-epimerase involved in capsule synthesis. The gene encoding l-lactate permease (*lldP*) also remained downregulated. Additionally *floA*, encoding scaffold protein flotillin and a gene encoding an acyltransferase were also downregulated. Genes *recR* and *rinB* remained significantly upregulated, as did *sspA*, and genes for thiamine and vitamin B6 metabolism. Seven genes involved in mechanistic components of translation were also upregulated while one was downregulated (Fig. 5).

#### Twenty-four hour timepoint

Eighty-eight significantly and differentially regulated genes were identified at the 24H timepoint. These included 31 downregulated and 57 upregulated genes in the *S. aureus*-mycolactone treatment compared to control. Forty-four genes associated with metabolism were differentially regulated (Fig. 5). Eight genes involved in amino acid metabolism were upregulated including members of the histidine utilization pathway (*hutU*, *hutG*, *hutI*), those for arginine and proline metabolism (*gdhA*, *rocD*, *pruA*, *putA*), and for aspartate family biogenesis (*lysC*). The only downregulated gene in this category was *trxB* (thioredoxin reductase). Both *lip1* and *lip2,* encoding lipases were upregulated. And, genes involved in general (*pnbA*, and a gene encoding diapolycopene oxygenase, and *pfla*, pyruvate formate lyase activating enzyme), menaquinone (*menC*) thiamine (*thiD)*, and vitamin B6 (*pdxS*) metabolism were also upregulated, while a gene for nitric oxide deoxygenase (*hmp*) and a gene for ribonucleoside-diphosphate reductase subunit beta (*nrdF*) was downregulated. Two genes for energy metabolism were upregulated including a gene encoding ATP F0F1 synthase subunit beta, and *yrp,* a nitronate monooxygenase. Downregulated genes involved in energy metabolism included *narZ* and *arcC*, both involved in nitrogen metabolism. Finally, twenty genes involved in carbohydrate metabolism were modulated. These included 10 genes downregulated involved in glycolysis or gluconeogenesis, the pentose phosphate pathway and anaerobic respiration and ten upregulated genes involved in gluconeogenesis or the TCA cycle.

Twenty-nine genes were differentially regulated that are known to be part of genetic information processing. These included downregulation of both copies of the cold shock protein (c*spC*), *hup,* involved in DNA replication and repair, *csbD*, involved in stress response, and 3 genes involved in transcription and translation mechanics. The remaining genes were upregulated. One is known to be involved in DNA repair (r*ecR*), seven are chaperones (*dnaJ, dnaK, groEL, groES, grpE, clpX and clpB*), three are transcriptional regulators (*rinB*, *ctsR* and *hrcA*), one encodes a general stress protein (*yzzA*), and two are involved in ribosome biogenesis.

Three virulence genes were upregulated, including clumping factor a (*clfA*), serine-aspartate repeat-containing protein C (*sdrC*), and surface protein F (*sasF*) while surface protein G (*sasG*) was downregulated. Five genes involved in signaling and cellular processes were modulated, including downregulation of *ptr2*, encoding a peptide ABC transporter permease and a LysM peptidoglycan-binding domain-containing protein. However*, sspA*, UDP-N-acetylglucosamine-peptide N-acetylglucosaminyltransferase GtfA subunit (*gtfA*)t, and a phosphate starvation-inducible protein PhoH (*phoH*) were upregulated. Seven genes were modulated involved with environmental information processing including upregulation of *mcsA* and *mcsB*, both part of the clpC operon and required for stress tolerance as well as upregulation of the alkaline shock response membrane anchor protein AmaP (*amaP*)^30^. Genes downregulated in this category included those involved in zinc transport (*znaB* and *znaC*), nitrate transport (*narT)*, and an autolysin (*aaa*). Finally, two uncharacterized genes were differentially upregulated at the 24H timepoint (Fig. 5).

## Discussion

Despite the array of *S. aureus* virulence factors, coinfection of *S. aureus* in an infected lesion from a BU patient does not elicit a pathological response typical to *S. aureus* wound infections. Studies have shown that QS and other secondary metabolite molecules of one bacterium can serve positive and negative regulatory roles in cell-to-cell communication in unrelated organisms^31^. Additionally, macrolide antibiotics have been shown to act as QS, virulence gene and biofilm antagonists at subinhibitory concentrations^32–36^. Mycolactone is a polyketide derived macrolide produced by MU that is also structurally similar to QS compounds in other bacteria, leading us to determine whether mycolactone attenuates virulence of other pathogenic bacteria that may co-colonize with MU during skin infection.

Our RT-qPCR data show that mycolactone downregulates *S. aureus* global response regulators *saeR* and *agr*A, as well as *hla* in a dose dependent manner. We have also shown that mycolactone attenuates hemolytic activity, and suppresses *hla* promoter activity. Further, mycolactone elicits these responses without inhibiting *S. aureus* growth, except at very high, non-clinically relevant concentrations^3,37^. This mycolactone targeting of genes and cellular processes responsible for pathogenesis and virulence rather than those necessary for growth are expected to impose a less restrictive selective pressure on *S. aureus*. It will thus be interesting to dig deeper into whether MU uses mycolactone for targeting of *S. aureus* social activities within a wound, and how that might impact microbial community ecology and resulting pathologies within that context.

The Agr system is responsible for expression of over 70 *S. aureus* genes, including many secreted virulence factors such as leukocidins, enterotoxins, lipases, and exoproteases and is also responsible for detachment of biofilms^38–42^. SaeR is the response regulator that is part of the major Sae global regulator system of many *S. aureus* virulence genes^43^. The Sae system regulates the expression of α-toxin by binding to the consensus SaeR-binding site upstream of the *hla* promoter, though *hla*, as well as other genes regulated by Sae can also be regulated by multiple regulators containing the SaeR binding sequence, such as the P1 promoter of both *SaeRS* and *hla* to promote autoinduction (via P1 promoter) and virulence^21,44–46^. Despite our findings, mechanisms by which mycolactone suppresses transcription of the *hla* gene remain elusive, though studies are underway.

Mycolactone diffuses rapidly and passively through the plasma membrane within target eukaryotic cells^47–49^. Besides cytopathic effects, mycolactone blocks co-translational translocation of inflammatory mediators through direct interaction with the α-subunit of the Sec61 secretory system, resulting in protein translation in the cytosol where they are degraded by the proteasome, and a lack of inflammatory infiltrates in ulcerative lesions^5,6^. Similar secretion systems such as SecYEG are present in prokaryotes, which are responsible for secretion of several proteins^50^. In *S. aureus,* these secretion systems are involved in secretion of several toxins and virulence factors^51^. So, the question arises of whether the effect of mycolactone on protein secretion is limited to eukaryotes or does it also extend to prokaryotes? Our RNASeq data showed the gene encoding preprotein translocase subunit SecY was significantly downregulated. This protein is part of the protein translocation channel required for secretion of some exported proteins beyond the cytoplasm to the cell surface or to secrete proteins out of the bacterium^52–54^. The gene *floA*, encoding flotillin that assists in assembly of membrane components and in the type VII secretion system was also downregulated^55^. These data suggest that mycolactone might also be blocking protein secretion pathways in *S. aureus.* Though this requires further investigation.

Carbohydrate and energy metabolism, particularly in gluconeogenesis and the tricarboxylic acid (TCA) cycle, were also expressed at a greater level in our *S. aureus* samples incubated with mycolactone compared to controls. Phosphoenolpyruvate (PEP) is the substrate for gluconeogenesis, generated from the intermediates of the tricarboxylic acid (TCA) cycle. Additionally, genes for arginine, proline and aspartate family biosynthesis were upregulated, as were genes for histidine catabolism. These results suggest a metabolic flux where *S. aureus* was differentially regulating the flow of nitrogen and carbon through the cell in response to mycolactone. Also, the arginine pathway has been shown to be important for *S. aureus* persister cell formation for antibiotic and stress tolerance as well as successful survival on host skin during infection^56,57^.

The amphipathic nature of mycolactone suggested that it may also exert its activity against *S. aureus* by perturbing membrane function and has other toxic effects leading to induction of genes involved in stress response. Indeed, a recent study outlined mycolactone’s preference for membrane relative to aqueous environments^47^. Furthermore, our RNASeq data point to signs of significant response to mycolactone supplementation. Lipases *lip1* and *lip2* were upregulated. Interestingly, *fakb2* that preferentially binds unsaturated fatty acids for their uptake was also upregulated^58^. Stress genes *mcsAB* and the alkaline stress response gene *amaP* were upregulated. Also, known cell wall stress stimulon members including *clpC*, *clpB*, and *clpX*, in addition to genes encoding the major heat shock proteins *GroE*L, *GroES*, *DnaK*, and *DnaJ* had significantly increased expression. Treatment of *S. aureus* with cell wall-active antibiotics is believed to result in the accumulation of damaged, misfolded, and aggregated proteins, and is also likely to be the same in the presence of mycolactone^59,60^. But, *hrcA* and *ctsR* encoding proteins that negatively regulates gene expression of these loci were also upregulated^61^. This paradoxical upregulation has been found in another study where the authors suggested an explanation that the CtsR repressor needs ClpC protein to be active^61^. Also, mycolactone induced metabolic flux, which may influence the availability of intracellular ATP levels required for repression and impact transcriptional control of both CtsR- and HrcA operons.

Our RNASeq data also showed *hla*, *agrA*, and *SaeR* genes were modulated across timepoints (Supplemental Table S1). However, these data were not statistically significant. And while the RNASeq data presented here give plausible explanation for transcriptome differences between *S. aureus* wild-type and mycolactone supplemented treatments, future studies with increased replication will elucidate these processes in more detail. Future work will also include analyses of the *S. aureus* secretome during mycolactone supplementation.

Presence of other bacteria such as *S. aureus*, and other pathogens with virulence potential, isolated from BU wounds could contribute to the delay in wound healing of patients, especially following the initiation of antibiotic treatment against MU^26^. However, without studies on *S. aureus* virulence factor expression and other microorganisms, within or isolated from BU wounds, as well as host response within this context, the role of *S. aureus* in delaying wound healing has only been presumptive. Within the wound, it is therefore important to understand the interaction of MU with skin flora and other wound residents in determining MU infection and pathology, as well as to measure local immune response to co-infection. This is also important in determining treatment outcomes of BU following antibiotic treatment where MU replication is slowed or ceased^62^. Only three isolation studies have been conducted on *S. aureus* contamination in BU wounds, but have reported *S. aureus* in 14.5% to 63.3% of BU wounds^9,10,63^, indicating an urgent need to understand these interactions.

Amissah et al. isolated *S. aureus* from 30 BU patients. From these, 26% of the isolates showed the same *S. aureus* genotype from individual patients’ wound and nose, but with *agr*-type diversity; many of these isolates were also resistant to clinically relevant antibiotics^9^. *S. aureus* colonizing BU wounds belonged to *agr* types II, III and IV, which are responsible for diseases such as toxic shock syndrome and Staphylococcal Scalded Skin Syndrome due to production of TSST-1 and exfoliatin respectively^26,64^. A recent study of 21 of those *S. aureus* isolates showed temporal changes in *S. aureus* genotypes in the wounds of some BU patients, with some *S. aureus* genotypes containing additional virulence genes over time, though all harbored core virulence genes^26^. It was also interesting in that study, that, in many of the *S. aureus* isolates, α-, β- and δ-hemolysin genes were present, though their activity was only detected in some isolates^26^. It was hypothesized that the lack of hemolytic activity could be due to a suppression of *agr* function by upstream regulators, such as *sigB;* however, this was not assessed in that study^26^.

Our data of mycolactone and *S. aureus* sensing and response at the transcriptional levels provide initial insights into biological mechanisms of interspecific interactions that may play a role in regulation of responses such as effects between MU, mycolactone, and *S. aureus* virulence, gene expression, proteome and exometabolome, toxin production, and MU-specific QS antagonism. These data also have implications for microbial interactions with MU and other microbes, and mycolactone mechanisms for MU fitness in natural environment and host niches. For instance, it is well known that changes in microbiomes can promote resistance to or infection by pathogenic bacteria. Also, our data could suggest an evolutionary role of this chemical cross-talk in shared MU and staphyloccal natural and host environments that merits further investigation. Our study also describes how a pathogen can modulate regulatory signals derived from skin microbiota (normal flora and those with pathogenic potential), further defining interspecies interactions. Results of these data suggest that BU pathology may, in part, be the result of multispecies synergistic and antagonistic interactions, further increasing disease complexity. More broadly, acute and chronic wound infections are a significant health problem around the world. And, data evaluating the roles of microorganisms and their specific interactions, as well as host immune response in this context, will have consequences for understanding disease pathogenesis, wound healing, and better patient management, as this knowledge is critical for successful management of wounds.

## Materials and methods

### Overall approach

**Figure 6:**
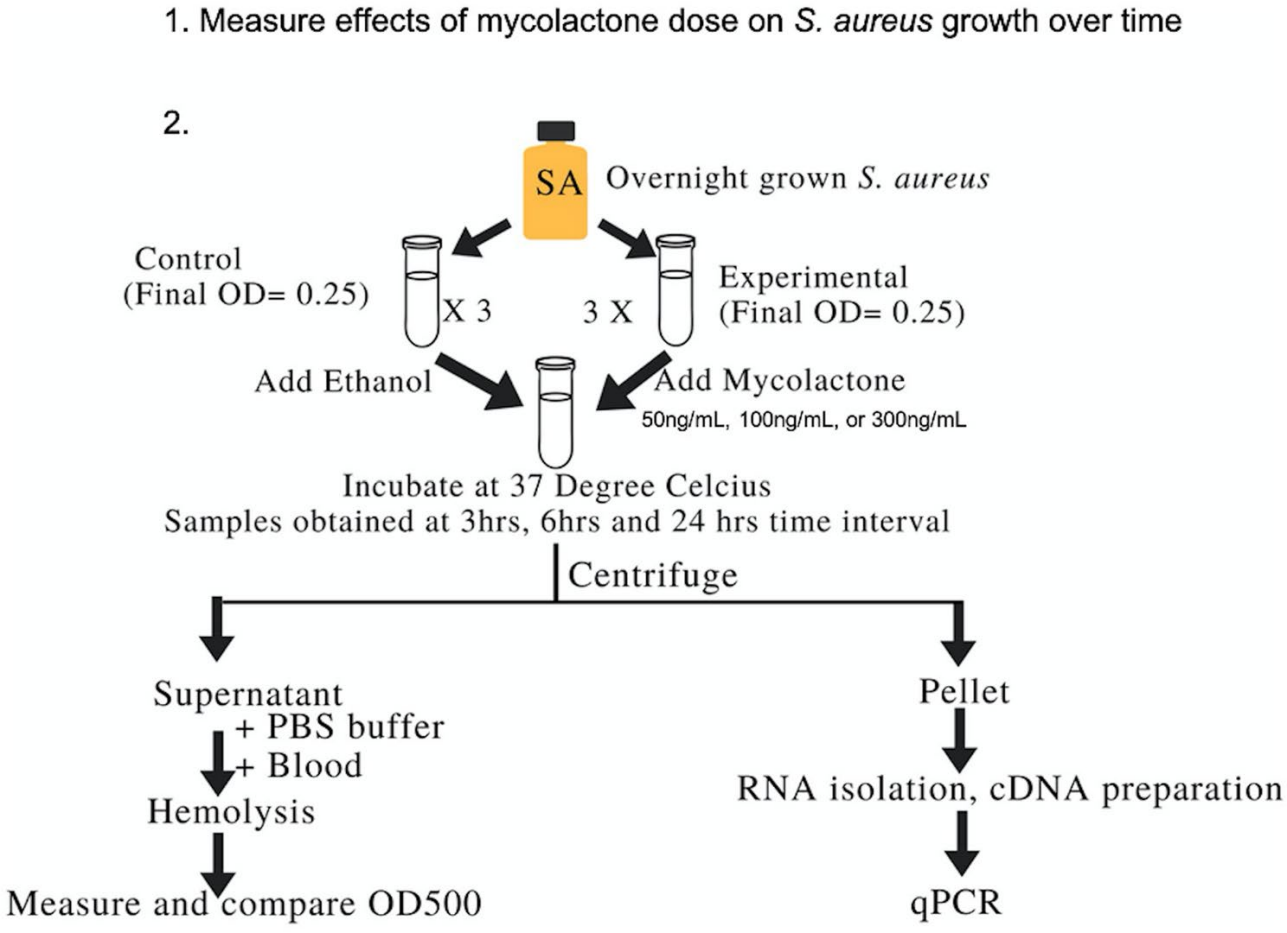
Overall approach to determine the effect of mycolactone on *S. aureus* growth, hemolytic activity and virulence gene expression.

Overnight grown *S. aureus* 502a^65^ was transferred to Luria Bertani (LB) broth such that the final OD600 was 0.25. Synthetic mycolactone A/B (donated from Yoshito Kishi’s laboratory, Department of Chemistry and Chemical Biology, Harvard University) was diluted to concentrations of 50 ng, 100 ng and 300 ng/mL in ethanol, dried down, then added in a 5 μL volume (in ethanol, EtOH) to respective flasks containing *S. aureus*, then incubated at 37 °C. Ethanol (5 μL) was also added to separate flasks containing *S. aureus* as a solvent control. At 3H, 6H, and 24H of incubation, samples were taken for measurement of growth (OD600), hemolytic activity, and relative quantitation of *agrA, saeR* and *hla* gene expression (Fig. 6). Experiments were performed at least three times and in sample triplicate.

### Measurement of *S. aureus* hemolytic activity

Hemolytic activity of *S. aureus* incubated with mycolactone was measured and compared with *S. aureus* containing ethanol (*S. aureus* Alone) using a modified pneumolysin hemolysis assay^66^. Briefly, dilution buffer containing phosphate buffered saline (PBS) and bovine serum albumin (BSA) was prepared and added to a 96 well V-bottom plate. *S. aureus* supernatant was filtered through a 0.2-micron filter and 100 μL was added to respective wells and serially diluted in a 1:2 dilution. Fifty microliters of PBS washed rabbit blood was added to each well and incubated at 37 °C for 1H. Distilled water was used as a positive control for hemolytic activity and buffer was used as a negative control. After incubation, the plate was centrifuged at 1000×*g* and supernatant was carefully transferred to a 96 well flat bottom plate where OD540nm was measured to determine the hemolytic activity of *S. aureus* incubated with mycolactone or EtOH, relative to the distilled water + red blood cell lysis positive control and to *S. aureus* + ethanol control.

### RNA isolation

RNA was isolated using the Trizol method according to manufacturer’s instructions. Briefly, bacterial cell suspensions were centrifuged and Trizol reagent was added to the pellet, mixed thoroughly, and homogenized with 0.2 mm beads. After incubation, chloroform was added and centrifuged for phase separation. The aqueous phase containing RNA was obtained and precipitated using isopropanol followed by washing with 75% ethanol. The pellet was dried and dissolved in nuclease-free water to obtain RNA suspension. The RNA was cleaned using the Qiagen RNeasy PowerClean Pro Cleanup Kit. RNA quality was analyzed by agarose gel electrophoresis and RNA concentrations were determined using Qubit 2.0. RNA was treated with Turbo DNAse (Invitrogen) according to the manufacturer’s instructions, to remove trace DNA as necessary. All samples were stored at − 80 °C until further processing for cDNA preparation and RT-qPCR, or for library preparation (described below).

### *S. aureus* cDNA preparation

*Staphylococcus aureus* cDNA was prepared using the Verso enzyme kit (Thermo Scientific) according to the manufacturer’s instructions. The reaction mixture for cDNA preparation included 4 μL synthesis buffer, 2 μL dNTP mix, 1 μL random hexamer, 1 μL Verso enzyme and 1 μL RT enhancer and the template. The reaction mixture was heated at 42 °C for 1H to obtain cDNA.

### Quantitative real time PCR (RT-qPCR)

RT-qPCR was performed targeting *agrA, saeR* and *hla* genes. The Shikimate dehydrogenase (*aroE*) gene was used as a housekeeping gene, and appropriate positive and negative controls were included in each run. The master mix contained 1.0 µL of each forward and reverse primer for *aroE* gene and target gene, 2.0 µL of target probe and *aroE* probe, 12.5 µL of master mix, 0.5 µL water and 3.0 µL template cDNA per well of PCR plate. PCR was conducted using a BioRad CFX96 with the following parameters: 95 °C for 10 min, and 39 cycles of 95 °C for 15 s, and 57 °C for 30 s for the *hla* gene and 39 cycles of 95 °C for 15 s or 59 °C for 30 s for *agr*A and *sae*R genes, respectively. The sequences of forward and reverse primers used for each gene are listed in Table 1.

**Table 1:** Primer sequences of genes amplified in RT-qPCR.

Primers	Sequence
Forward *aroE*	5′ATGGCTTTAATATCACAATTCC3′
Reverse *aroE*	5′CTATCCACTTGCCATCTTTTAT3′
Forward *agrA*	5′TCACAGACTCATTGCCCATT3′
Reverse *agrA*	5′CCGATGCATAGCAGTGTTCT3′
Forward *saeR*	5′GCTAAATACCACATAACTCAAATTCC3′
Reverse *saeR*	5′TTGAACAACTGTCGTTTGATGA3′
Forward *hla*	5′GTGTATGACCAATCGAAACATTTGCA3′
Reverse *hla*	5′GGTAATGTTACTGGTGATGATACAGGAA3′

### Library preparation, RNA seq and analysis

RNA libraries were created from combined triplicate RNA samples *of S. aureus* controls and *S. aureus* with mycolactone supplementation (300 ng/mL concentration) at the above timepoints. Libraries were created using the NEBNext^®^ Ultra™ RNA Library Prep Kit and NEBNext^®^ Multiplex Oligos (Dual Index Primers) for Illumina® and associated protocols. High-throughput RNA sequencing was performed by St. Jude Children’s Research Hospital on an Illumina HiSeq2000 with 2 × 150 bp PE (paired end) read lengths. Sequences are archived and publicly available within NCBI Sequence Read archive under submission number SUB8456005, and BioProject number PRJNA653874. Sequences were initially trimmed by the sequencing facility using TrimGalore v0.4.2^67^, but a more stringent quality trimming was also performed using default parameters within the Qiagen CLC Workbench 20.0.1 (https://www.qiagenbioinformatics.com/) following QC analysis of sequence reads. Resulting high-quality reads were aligned to the *S. aureus* NZ_CP007454 reference genome (downloaded from the NCBI database). RNASeq data were mapped with the following parameters: (a) maximum number of allowed mismatches was set at 2, with insertions and deletions set at 3; (b) Length and similarity fractions were set to 0.9, with autodetection for both strands; (c) minimum number of hits per read was set to 10. Differential expression was measured between *S. aureus-*mycolactone treatment against *S. aureus*-EtOH control for the 3H, 6H, and 24H timepoints in the CLC Workbench, that used the assumption that genes with similar average expression levels had similar variability, according to the CLC Manual. The program uses multi-factorial statistics based on a negative binomial generalized linear model. Treatment reads with an absolute fold change of 1.5 or higher, and FDR adjusted p-value less than or equal to 0.05 were considered significant. Transcripts were further annotated into pathways by linking protein ID with potential conserved domains and protein classifications archived within the Conserved Domain Database (https://www.ncbi.nlm.nih.gov/Structure/cdd/cdd.shtml), and by using the UniProt, KEGG and STRING databases^68–71^.

### Impact of mycolactone on hla promoter activity

To monitor *hla* promoter activity, the *hla* promoter was amplified and transcriptionally fused with the LuxABCDE operon in a pMK4 vector^72^. The luminescence reporter plasmid was electroporated into *S. aureus* LAC^73,74^ strain harboring the luminescence reporter plasmid and was cultured in brain heart infusion broth supplemented with 50–500 ng of mycolactone or vehicle control (DMSO) at 37 °C for 24H. The luminescence signal was monitored by Cytation 5 (BIoTek).

### Statistical analysis

Significant difference in growth, percent reduction in hemolysis, and *hla* promoter activity (luminescence) of *S. aureus* containing mycolactone compared to *S. aureus*-EtOH control was determined using one-way analysis of variance (ANOVA). The RT-qPCR data was analyzed using Python code implementing the ΔΔCT method to determine fold change relative to housekeeping control and significant difference (p-value < 0.05)^75^. Resulting regulation was determined relative to control samples, which was considered baseline. A fold change greater than 1 was considered as upregulated. For fold change between 0 and 1, the negative of the reciprocal of fold change was calculated to determine downregulation.

## Supplementary Information

Supplementary Legends.

Supplementary Figure S1.

Supplementary Figure S2.

Supplementary Table S1.

## Data Availability

The datasets generated during and/or analysed during the current study are available in the NCBI Sequence Read archive under BioProject number PRJNA653874 [https://www.ncbi.nlm.nih.gov/bioproject/PRJNA673874].
