# Supplementary material for: Responses to chemical cross-talk between the Mycobacterium ulcerans toxin, mycolactone, and Staphylococcus aureus
Source: Sci Rep. 2021 Jun 3;11:11746. doi: 10.1038/s41598-021-89177-5 (PMC8175560; doi:10.1038/s41598-021-89177-5)
Supplement: Supplementary file 1 — Supplementary Legends. [file 41598_2021_89177_MOESM1_ESM.docx]

**Supplemental Figure 1.** Promoter activity *hla* in culture supplemented with mycolactone (ML, 200 – 500 ng) or vehicle control (VC, EtOH), measured by a luminescent report gene.

**Supplemental Figure 2.** Volcano plot showing statistical significance (-log_10_ p-value) versus magnitude of transcription change (log_2_ fold change) in *S. aureus* + EtOH versus *S. aureus* + mycolactone at 3H, 6H, and 24H. Statistically significant differences are shown in red.

**Supplemental Table 1.** Complete gene lists of significantly up-or down-regulated genes according to timepoint.
