# Supplementary figures and images for: Responses to chemical cross-talk between the Mycobacterium ulcerans toxin, mycolactone, and Staphylococcus aureus
Source: Sci Rep. 2021 Jun 3;11:11746. doi: 10.1038/s41598-021-89177-5 (PMC8175560; doi:10.1038/s41598-021-89177-5)

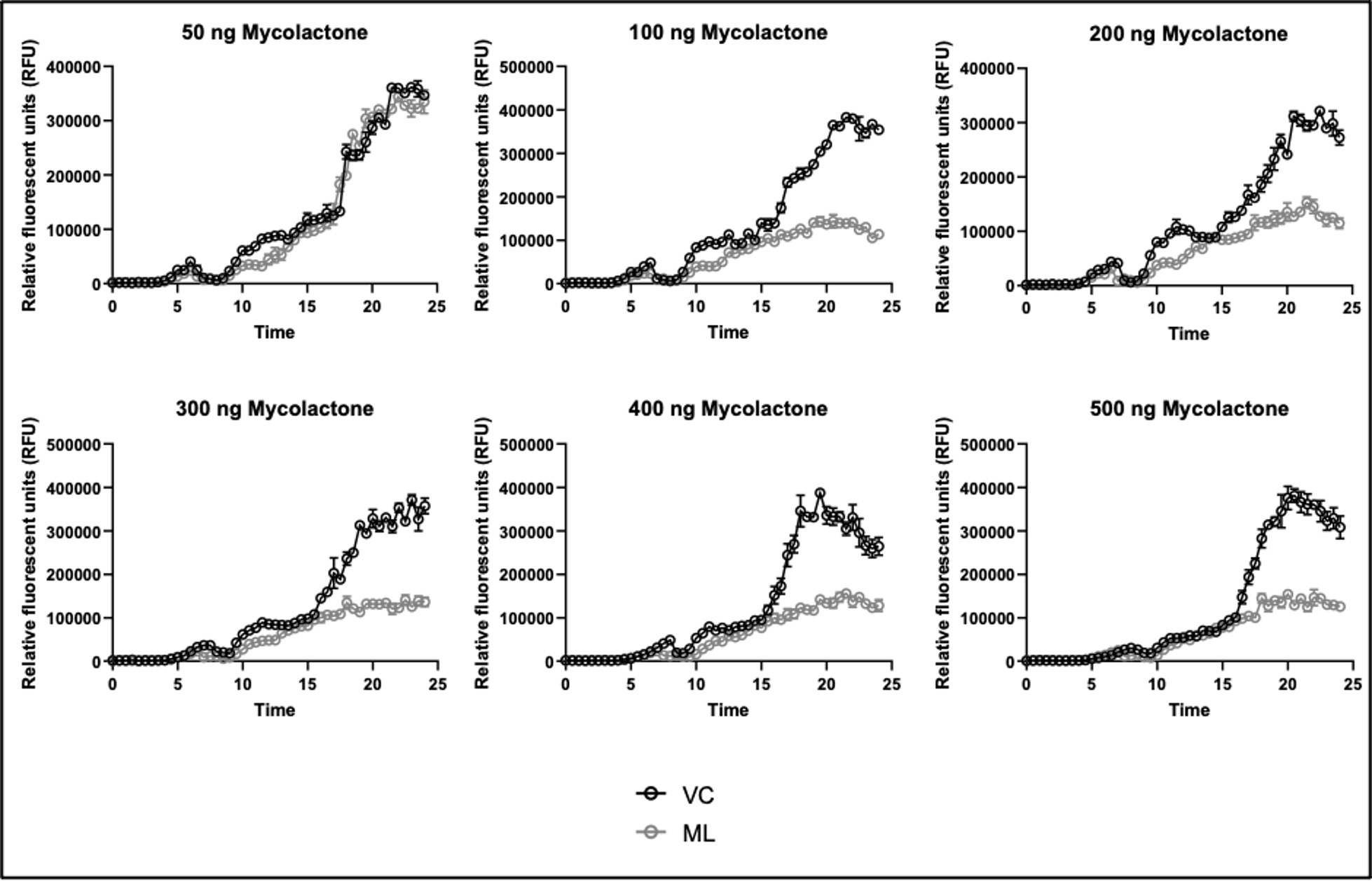

Supplement: Supplementary file 2 — Supplementary Figure S1. [file 41598_2021_89177_MOESM2_ESM.png]

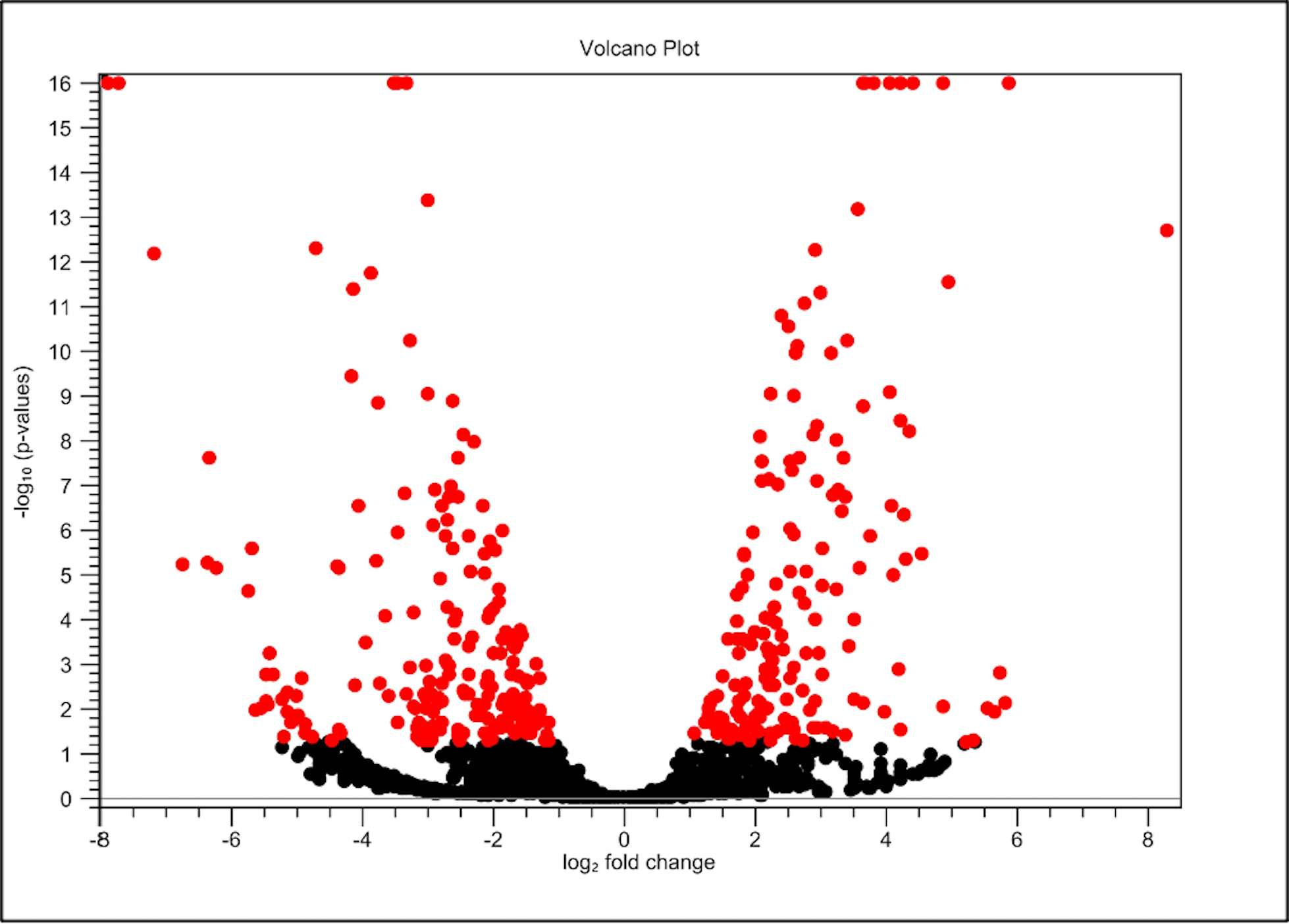

Supplement: Supplementary file 3 — Supplementary Figure S2. [file 41598_2021_89177_MOESM3_ESM.png]
